# Multiomics analyses unveil the involvement of microRNAs in pear fruit senescence under high- or low-temperature conditions

**DOI:** 10.1038/s41438-020-00420-y

**Published:** 2020-12-01

**Authors:** Chao Gu, Huan-Yu Xu, Yu-Hang Zhou, Jia-Long Yao, Zhi-Hua Xie, Yang-Yang Chen, Shao-Ling Zhang

**Affiliations:** 1 Centre of Pear Engineering Technology Research, State Key Laboratory of Crop Genetics and Germplasm Enhancement, Nanjing Agricultural University, Nanjing, 210095 China; 2 New Zealand Institute of Plant & Food Research Ltd., Private Bag 92169, Auckland, 1142 New Zealand

**Keywords:** Non-coding RNAs, Abiotic

## Abstract

Senescence leads to declines in fruit quality and shortening of shelf life. It is known that low temperatures (LTs) efficiently delay fruit senescence and that high temperatures (HTs) accelerate senescence. However, the molecular mechanism by which temperature affects senescence is unclear. Herein, through multiomics analyses of fruits subjected to postharvest HT, LT, and room temperature treatments, a total of 56 metabolic compounds and 700 mRNAs were identified to be associated with fruit senescence under HT or LT conditions. These compounds could be divided into antisenescent (I→III) and prosenescent (IV→VI) types. HT affected the expression of 202 mRNAs to enhance the biosynthesis of prosenescent compounds of types V and VI and to inhibit the accumulation of antisenescent compounds of types II and III. LT affected the expression of 530 mRNAs to promote the accumulation of antisenescent compounds of types I and II and to impede the biosynthesis of prosenescent compounds of types IV and V. Moreover, 16 microRNAs were isolated in response to HT or LT conditions and interacted with the mRNAs associated with fruit senescence under HT or LT conditions. Transient transformation of pear fruit showed that one of these microRNAs, Novel_188, can mediate fruit senescence by interacting with its target *Pbr027651.1*. Thus, both HT and LT conditions can affect fruit senescence by affecting microRNA–mRNA interactions, but the molecular networks are different in pear fruit.

## Introduction

Fruits contain a range of energy-providing molecules, nutrients, and bioactive compounds (including vitamins, minerals, and dietary fibers) that contribute to general health, decrease the risks of chronic diseases and thus are essential components of human diets^1^. Generally, fresh fruits are more popular among consumers than processed fruits. However, owing to large-scale production and intensive and time-consuming harvest processes, many fruits cannot be sold in a timely manner and thus must be stored to prolong the fruit supply period. During postharvest storage, fruit quality gradually deteriorates with ongoing metabolism; this process is called senescence. Senescence is the last phase of fruit development and accomplishes ordered disassembly of cell components and recycling of nutrients^2^. Senescent fruit is easily attacked by fungal pathogens that can trigger diseases accelerating fruit decay after harvest^3^.

During fruit senescence, a series of physiological and biochemical reactions that occur in stored fruits cause dramatic changes in texture, flavor, skin color, biochemical composition, and pathogen susceptibility^4^ that lead to declines in quality and nutrition. Thus far, several techniques have been developed to delay fruit senescence, such as low temperature (LT), 1-methylcyclopropene, calcium, controlled atmosphere, salicylic acid, ATPase, and oxalic acid treatments^5–7^. Of these techniques, temperature treatment is easy to perform and thus has been the most widely used to delay fruit senescence. Previous reports have shown that LT can retard firmness loss and slow metabolism during storage^8,9^, but the molecular mechanism of fruit senescence under LT conditions is still unclear.

In annual tomato, fruit senescence can be delayed by silencing *ACS2* (an ethylene biosynthesis gene) or overexpressing *FYFL* (a *MADS-box* gene)^10,11^. In perennial fruit trees, fruit senescence is negatively correlated with the expression patterns of energy-related genes in lychee^7^ and may be mediated by histone deacetylase HD2 and ethylene responsive factor ERF1 in longan^12^. In pear, 951 genes correlate to fruit senescence, and 77 genes are involved in ethylene-induced fruit senescence^13^. However, the molecular network regulating fruit senescence is also unclear in these fleshy fruits.

MicroRNAs are short noncoding RNAs consisting of 18–30 nucleotides^14^ and have been shown to be negative regulators of gene expression in eukaryotes. In this century, microRNAs have been widely studied in plants and are reported to be involved in root architecture, leaf and flower morphogenesis, developmental timing, fruit quality, and responses to biotic and abiotic stresses^15–19^. Moreover, microRNA–target interactions have been detected during fruit ripening and senescence based on an integrative analysis of the microRNAome and degradome^20,21^. However, little is known about the roles of microRNAs in fruit senescence.

Pear is a popular fruit and has been cultivated worldwide. In China, pear production is ~16.2 million tons (FAOSTAT 2018). Pear fruits are harvested in summer and show fast senescence because of high temperatures (HTs; >35 °C) in production areas. To prolong shelf life, the harvested fruits are usually stored at LT (~4 °C) to delay senescence. However, little is known about the molecular mechanism by which temperature affects senescence. In this study, pear fruits were stored at high, low, and room temperatures (RTs). Fruit samples that were collected at different stages of storage under the three different temperatures were analyzed to determine the levels of metabolic compounds, mRNAs, and microRNAs. Differential analysis of compounds was conducted to identify the compounds associated with fruit senescence under HT or LT conditions. These identified compounds were used as indexes to isolate the mRNAs associated with fruit senescence under HT or LT conditions by correlation analysis. Moreover, microRNA–target analysis was performed to determine how many of these mRNAs are the targets of the microRNAs expressed in pear fruits. Finally, a dual-luciferase assay was used to confirm microRNA–mRNA interactions, and transient transformation of pear fruit was used to unveil the roles of a microRNA (Novel_188) and its target (*Pbr027651.1*) in fruit senescence. This information is useful for optimizing postharvest handling methods in order to extend storage life and maintain fruit quality during storage.

## Results

### Experimental design and postharvest treatments

**Fig. 1:**
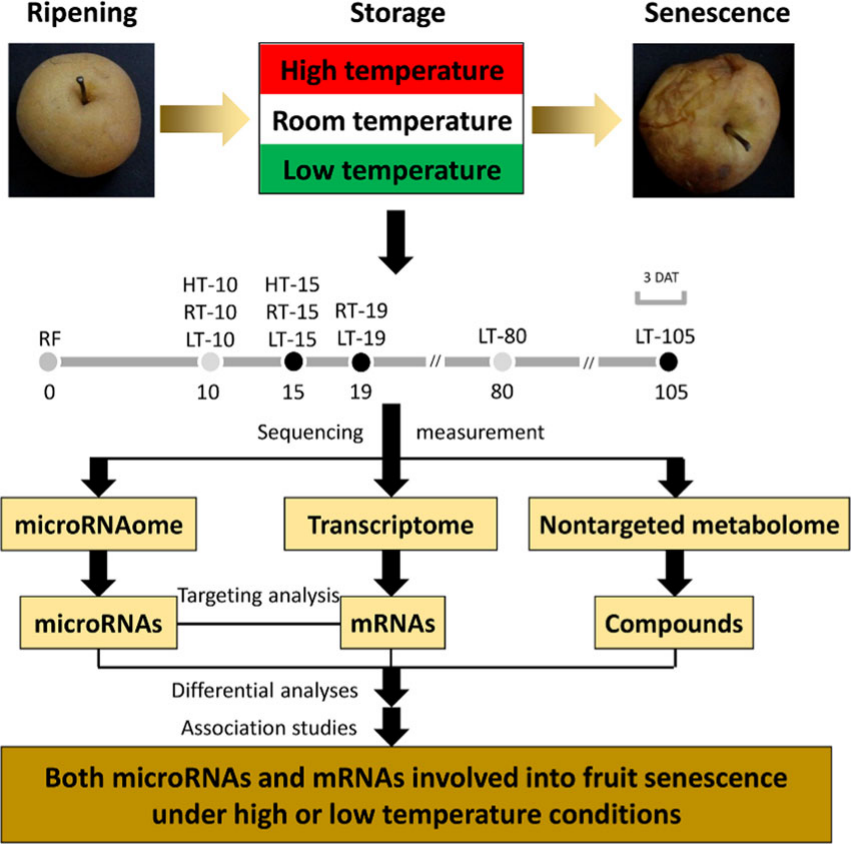
Experimental design for assessment of fruit senescence under different temperature conditions. Ripening fruits were harvested and then subjected to HT, LT, or RT (controls). During fruit senescence, fruit flesh samples were collected at 0, 10, 15, 19, 80, and 105 days after treatment (DAT). Notably, the fruits under the HT, RT, and LT treatments were senescent at 15, 19, and 105 DAT, respectively. The collected samples were used for a multiomics study of the nontargeted metabolome, transcriptome, and microRNAome. These data were used to clarify the molecular mechanism of fruit senescence under HT or LT conditions

To clarify the molecular networks of fruit senescence under different temperatures, harvested fruits were treated with HT and LT, and fruits with RT treatment were used as controls. The fruits stored at HT, RT, and LT were senescent at 15, 19, and 105 days after treatment (DAT), respectively (Fig. 1). Therefore, fruit flesh samples were collected at 0, 15, 19, and 105 DAT for multiomics studies of the microRNAome, transcriptome, and nontargeted metabolome (Fig. 1). To enhance the reliability of the sequencing data, fruit flesh samples were also collected at 10 and 80 DAT for a multiomics study. Differential analyses were performed between RF and senescent fruit samples (HT-15, RT-19, or LT-105), between HT-15 and RT-15 samples, and between RT-19 and LT-19 samples. Then, correlation analysis was conducted between the differential mRNAs and compounds using the data generated from the 11 groups of samples to identify the mRNAs responsive to fruit senescence under HT or LT conditions. Third, microRNA–target analysis was completed between microRNAs and mRNAs to clarify the microRNAs involved in fruit senescence under HT or LT conditions. Finally, the potential microRNA–mRNA interactions were tested by a dual-luciferase assay in tobacco leaves, and the roles of Novel_188 and its target *Pbr027651.1* were tested by transient transformation of pear fruit.

### Identification of the compounds associated with fruit senescence under HT or LT conditions

**Fig. 2:**
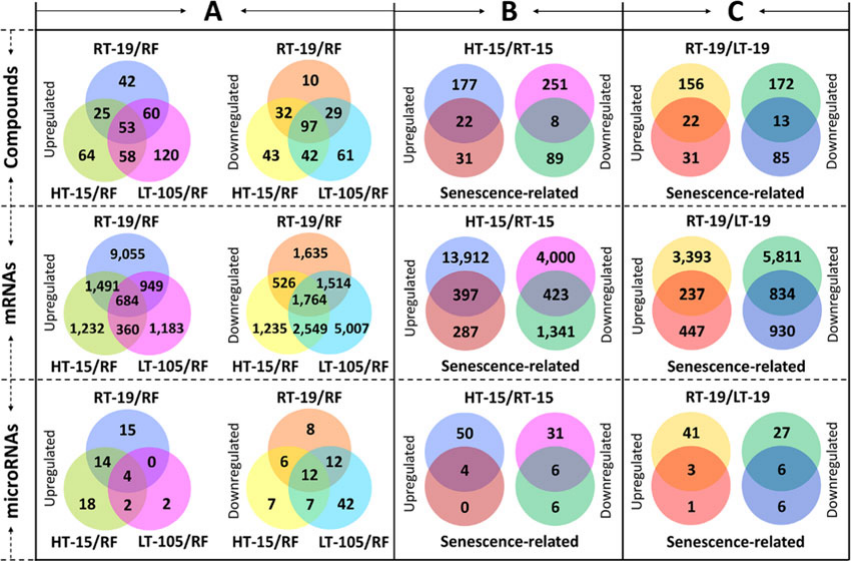
Differential analyses of compounds, mRNAs, and microRNAs. **a** Differential analyses were conducted between RFs and senescent fruits (RT-19/HT-15/LT-105) to identify the compounds/mRNAs/microRNAs responsive to fruit senescence. **b** Differential analyses were conducted between HT-15 and RT-15 (HT-15/RT-15) samples to identify the compounds/mRNAs/microRNAs responsive to HT and fruit senescence under HT conditions. **c** Differential analyses were conducted between RT-19 and LT-19 (RT-19/LT-19) samples to identify the compounds/mRNAs/microRNAs responsive to LT and fruit senescence under LT conditions

To identify the compounds associated with fruit senescence, high-throughput nontargeted metabolomic analysis was performed using the 11 groups of samples. A total of 2138 distinct metabolic compounds were detected in pear fruits (Table S1). Among these compounds, 53 exhibited higher levels and 97 exhibited lower levels in senescent fruit (HT-15, RT-19, and LT-105) samples compared with RF samples (Fig. 2a). Therefore, these 150 compounds are associated with fruit senescence.

To isolate the compounds associated with fruit senescence under HT conditions, differential analysis was performed between HT-15 and RT-15 (HT-15/RT-15) samples, which showed that 199 compounds exhibited higher levels and 259 exhibited lower levels in HT-15 compared with RT-15 samples (Fig. 2b). Among these differentially accumulated compounds, 30 overlapped with the compounds associated with fruit senescence (Fig. 2b and Table S2) and thus are associated with fruit senescence under HT conditions. These compounds included isobutylpropylamine (POS0032), gamma-coniceine (POS0044), 3-nonenal (POS0061), docosanedioic acid (POS0259), aragusteroketal (POS0435), and oscillatoxin A (POS0542).

Similarly, differential analysis was performed between RT-19 and LT-19 (RT-19/LT-19) samples, which showed that 178 compounds exhibited higher levels and 185 compounds exhibited lower levels in RT-19 compared with LT-19 (Fig. 2c). Among these differentially accumulated compounds, 35 overlapped with the compounds associated with fruit senescence (Fig. 2c and Table S3) and thus are associated with fruit senescence under LT conditions. These compounds included 1-hexadecylamine (POS0147), 2-chlorohexadecanol (POS0164), palmitic amide (POS0189), C16 sphinganine (POS0218), docosanedioic acid (POS0260), monogalactosyl (POS0288), succinic acid semialdehyde (NEG0045), stanozolol (NEG0412), and 5,10-methylene-THF (NEG0553).

Notably, only nine compounds, including 5,10-methylene-THF (NEG0550) and phosphatidylserine (NEG0551), were differentially accumulated between HT-15/RT-15 and RT-19/LT-19 samples (Tables S2, S3) and thus are associated with fruit senescence under HT and LT conditions. This result suggests that physiological mechanisms of fruit senescence that operate under both HT and LT conditions are relatively uncommon.

### Identification of the mRNAs associated with fruit senescence under HT or LT conditions

To identify the mRNAs associated with fruit senescence, high-throughput analysis of the transcriptome was performed using the 11 groups of samples. A total of 271.9 million reads were mapped to 34,096 mRNAs (Table S4). Among these mRNAs, 684 were upregulated and 1764 were downregulated in senescent fruits compared with RFs (Fig. 2a). Therefore, these 2448 mRNAs may be involved in fruit senescence.

**Fig. 3:**
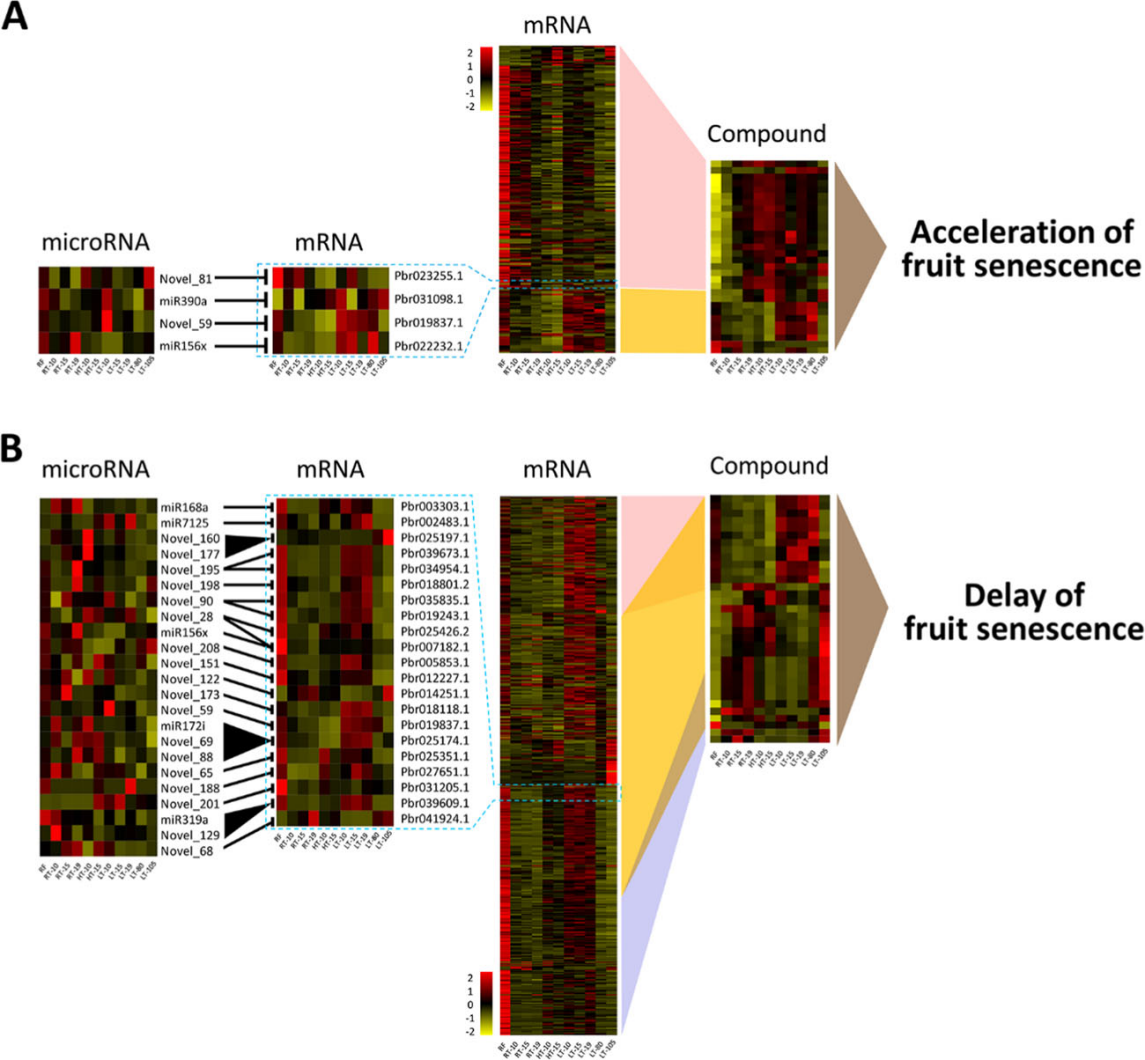
Diagram of the microRNA–mRNA-compound pathway during fruit senescence under HT or LT conditions. **a** During fruit senescence under HT conditions, HT-responsive mRNAs could be divided into two groups that were correlated to HT-induced (linked with a red box) and HT-repressed (linked with a yellow box) compounds. Of these mRNAs, four were the targets of four microRNAs. miR390a was positively responsive to HT conditions, while miR156x was negatively responsive to HT conditions. Neither Novel_59 nor Novel_81 was responsive to HT conditions. **b** During fruit senescence under LT conditions, LT-responsive mRNAs could be divided into three groups that were correlated to 13 (linked with a red box), 32 (linked with a yellow box) and 10 (linked with a blue box) HT-responsive compounds. Of these mRNAs, 21 were the targets of 23 microRNAs. Five microRNAs, miR7125, Novel_122, Novel_129, Novel_188, and Novel_201, were positively responsive to LT conditions. Nine microRNAs, miR156x, miR168a, Novel_68, Novel_88, Novel_90, Novel_177, Novel_195, Novel_198, and Novel_208, were negatively responsive to LT conditions. The fold change is the ratio of the RPKM value in each sample, and the mean of all 11 samples was used for calculation of the log_2_(fold change) in the color bar

To isolate the mRNAs associated with fruit senescence under HT conditions, differential analysis was performed between HT-15 and RT-15 samples, which showed that 14,309 mRNAs were upregulated and 4423 mRNAs were downregulated in HT-15 compared with RT-15 samples (Fig. 2b). Among these differentially expressed mRNAs, 820 overlapped with the mRNAs involved in fruit senescence (Fig. 2b). However, correlation analysis showed that only 202 mRNAs correlated to all 30 compounds associated with fruit senescence under HT conditions (Table S5). Of these 202 mRNAs, 156 correlated to the 20 compounds positively responsive to fruit senescence under HT conditions (linked with a red box), whereas 46 correlated to the remaining 10 compounds (linked with a yellow box) that contained the eight compounds negatively responsive to fruit senescence and the two compounds (POS1162 and POS1166) positively responsive to fruit senescence under HT conditions (Fig. 3a). Therefore, these 202 mRNAs are involved in fruit senescence under HT conditions.

To isolate the mRNAs associated with fruit senescence under LT conditions, differential analysis was performed between RT-19 and LT-19, which showed that 3630 mRNAs were upregulated and 6645 mRNAs were downregulated in RT-19 compared with LT-19 samples (Fig. 2b). Among these differentially expressed mRNAs, 1071 overlapped with the mRNAs involved in fruit senescence (Fig. 2b). However, correlation analysis showed that only 530 mRNAs correlated to 34 of the 35 compounds associated with fruit senescence under LT conditions (Table S6). Of these 530 mRNAs, 277 correlated to all compounds except POS1162 and POS1166 (linked with the yellow box), 115 correlated to 13 compounds excluding POS1162 and POS1166 (linked with the red box), and the remaining 138 correlated to 10 compounds including POS1162 and POS1166 (linked with the blue box; Fig. 3b). Therefore, these 530 mRNAs are involved in fruit senescence under LT conditions.

Notably, only 32 mRNAs that were negatively responsive to fruit senescence were differentially expressed between HT-15/RT-15 and RT-19/LT-19 and thus are associated with fruit senescence under HT and LT conditions. This result suggests that the molecular mechanisms of fruit senescence under HT and LT conditions are different. In addition, quantitative reverse transcription PCR (RT-qPCR) validation showed that the expression profiles of nine randomly selected mRNAs were positively correlated to the profiles identified from the transcriptome data (*P* < 0.05; Figure S1), which indicated that the qRT-PCR results were consistent with the transcriptome data.

### Chromosomal location assessment and functional annotation

To unveil the roles of the genes involved in fruit senescence under HT or LT conditions, the chromosomal locations of these genes were first analyzed. Six genes that were positively responsive to fruit senescence under HT conditions were found to be individually distributed on chromosomes 3, 6, 7, 11, 13, and 17 (Figure S2). Among the 196 genes that were negatively responsive to fruit senescence under HT conditions, 173 were distributed on all 17 chromosomes, and 23 were distributed on 19 unanchored scaffolds (Figure S2). In contrast, among the 444 genes that were positively responsive to fruit senescence under LT conditions, 392 were distributed on all 17 chromosomes, and 52 were distributed on 45 unanchored scaffolds (Figure S3). Of the 86 genes that were negatively responsive to fruit senescence under LT conditions, 73 were distributed on most chromosomes except Chr14, and 13 genes were distributed on 14 unanchored scaffolds (Figure S3).

Functional annotation showed that one *Aux/IAA* and 14 Auxin-responsive *SAUR* genes (including *Pbr042215.1*) were negatively responsive to fruit senescence under HT conditions, whereas three *Auxin Efflux Carrier* (AEC), two *Aux/IAA* and two Auxin-responsive *SAUR* genes (also including *Pbr042215.1*) were positively responsive to fruit senescence under LT conditions (Table 1). This result indicates that auxin signaling is involved in fruit senescence under different temperature conditions. This conclusion is supported by previous reports that exogenous auxin can delay the ripening process of strawberries after harvest and reduce calyx senescence in *Citrus* fruits^22^.

**Table 1:** Functional annotations of the candidate genes

Treatment	Classification	Gene ID	Note
HT	AUX/IAA	Pbr004358.1	Negative
	SAUR	Pbr009913.1, Pbr009918.1, Pbr009920.1, Pbr009925.1, Pbr009929.1, Pbr023255.1, Pbr023257.1, Pbr040963.1, Pbr042208.1, Pbr042211.1, Pbr042213.1, Pbr042214.1, Pbr042216.1, Pbr042215.1	Negative
	MADS-box	Pbr005989.1, Pbr026075.1	Negative
	GRAS	Pbr017879.1, Pbr027642.1	Negative
	WRKY	Pbr008639.1	Negative
	ZFP	Pbr007236.1	Negative
	B3	Pbr019382.1	Negative
	NAC	Pbr001259.1	Negative
LT	POD	Pbr026235.1, Pbr031894.1	Positive
	CBF (AP2/ERF)	Pbr003581.1	Positive
	AEC	Pbr013424.1, Pbr013430.1, Pbr030776.1	Positive
	AUX/IAA	Pbr009069.1, Pbr025290.1	Positive
	SAUR	Pbr009912.1, Pbr042215.1	Positive
	MADS-box	Pbr018801.2	Positive
	GRAS	Pbr033535.1	Positive
	WRKY	Pbr004885.1	Positive
	ZFP	Pbr012907.1, Pbr019090.1, Pbr023221.1	Positive
	B3	Pbr000415.1, Pbr027478.1	Positive
	NAC	Pbr001259.1, Pbr015549.1, Pbr019213.1, Pbr026635.1,	Positive
	SBP	Pbr002303.1, Pbr007182.1	Positive
	ERF (AP2/ERF)	Pbr025174.1, Pbr025944.1	Positive
	bHLH	Pbr006330.2, Pbr013792.1, Pbr016539.1, Pbr041849.1	Positive
	HB	Pbr007308.1, Pbr016430.1, Pbr033829.1, Pbr033866.1	Positive
	bZIP	Pbr018746.1	Positive
	TCP	Pbr039609.1	Positive
	K-box	Pbr020189.1	Positive
	MYB	Pbr016796.1, Pbr041921.1	Positive

Moreover, six different types of transcription factors (TFs), MADS-box, GRAS, WRKY, zinc finger protein (ZFP; C_2_H_2_-type), B3, and NAM/ATAF1/2/CUC2 (NAC) TFs, were negatively responsive to fruit senescence under HT conditions and positively responsive to fruit senescence under LT conditions (Table 1). This result suggests that these six types of TFs are probably involved in fruit senescence under different temperatures. This conclusion is consistent with previous reports indicating that leaf senescence is negatively regulated by WRKY, ZFP, and NAC TFs^23–25^. Notably, an NAC TF (Pbr001259.1) was detected among the genes involved in fruit senescence under both HT and LT conditions, indicating that this NAC is associated with temperature-affected fruit senescence.

In addition, eight other different types of TFs, SBP, AP2/ERF, bHLH, HB, bZIP, TCP, K-box, and MYB TFs, were positively responsive to fruit senescence under LT conditions (Table 1). Of these types of TFs, CBF is reported to be induced by LT^26^. A MADS-box TF, SlFYFL, can delay leaf and flower senescence^11^. Thus, fruit senescence under different temperature conditions is mediated by multiple types of TFs.

### microRNA–mRNA interactions

To identify the microRNAs associated with fruit senescence, high-throughput analysis of small RNAs was performed using the 11 groups of samples. Approximately 64.15% of the total 122.39 million reads were mapped to 48 known and 119 novel microRNAs (Table S7). Among these microRNAs, Novel_59 could target the mRNA associated with fruit senescence under HT and LT conditions, and miR156x could target two different mRNAs that were associated with fruit senescence under HT and LT conditions (Fig. 3a, b). Moreover, two microRNAs, miR390a and Novel_81, could target the two mRNAs associated with fruit senescence under HT conditions (Fig. 3a). In contrast, the remaining 23 microRNAs could target the 20 mRNAs associated with fruit senescence under LT conditions (Fig. 3b). These results suggest that microRNA–mRNA interactions probably occur during fruit senescence under HT and LT conditions.

**Fig. 4:**
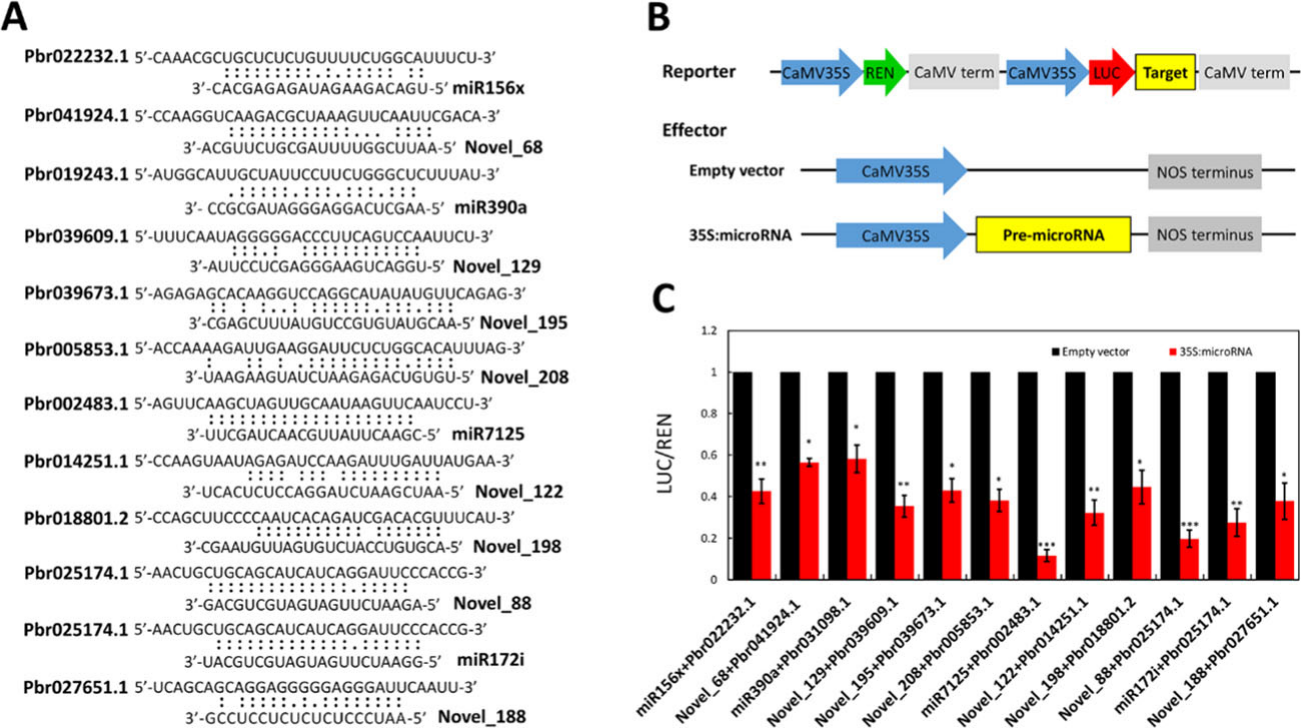
Validation of the predicted interactions between microRNAs and mRNAs. **a** Predicted interactions between microRNAs and their target sequences. **b** Construction of reporter and effector vectors. **c** Dual-luciferase assay of microRNA–target interactions. The means and standard errors were compared using Student’s *t* test. Single, double, and triple asterisks indicate significant differences at *P* values of < 0.05, < 0.01, and < 0.001, respectively

To test the potential microRNA–mRNA interactions, 12 pairs of microRNAs and mRNAs were selected for a dual-luciferase assay (Fig. 4a). A reporter was constructed by inserting target sequences into the 3’-flanking region of *LUC*, whereas the precursor of each microRNA was inserted into the enzyme sites of a pSAK277 vector to construct a 35S-derived pre-microRNA (35 S:microRNA; Fig. 4b). After transfecting the reporter and an effector construct together into tobacco leaves, we found that *LUC* activity in leaves cotransfected with the reporter and 35 S:microRNA was lower than that in leaves cotransfected with the reporter and an empty vector (Fig. 4c). This result suggests that the isolated microRNAs participate in fruit senescence under HT and LT conditions via microRNA–mRNA interactions.

### Identification of the microRNAs responsive to HT or LT conditions

To clarify whether the above microRNAs are responsive to different temperature conditions, differential expression analysis was first performed between senescent fruits and RFs. The results revealed that four microRNAs were upregulated and 12 microRNAs were downregulated in senescent fruits compared with RFs (Fig. 2a). Second, 54 microRNAs were upregulated and 37 microRNAs were downregulated in HT-15 compared with RT-15 samples (Table S8). The overlapping 10 microRNAs did not include any of the four microRNAs that could target the mRNAs involved in fruit senescence under HT conditions (Fig. 2b). This result indicates that these four microRNAs have expression profiles that differ from those of their targets. Interestingly, miR390a was upregulated and miR156x was downregulated in HT-15 compared with RT-15 samples (Fig. 3a), indicating that miR390a was positively responsive and that miR156x was negatively responsive to HT conditions. Therefore, HT can affect fruit senescence by mediating the expression of miR156x and miR390a.

Similarly, differential expression analysis showed that 44 microRNAs were upregulated and 33 microRNAs were downregulated in RT-19 compared with LT-15 samples (Table S9). Of these 77 microRNAs, nine were also differentially expressed between senescent fruits and RFs (Fig. 2c). These nine microRNAs did not include any of the 23 microRNAs that could target the mRNAs involved in fruit senescence under LT conditions. This result indicates that these 23 microRNAs have expression profiles that differ from those of their targets. Among these 23 microRNAs, miR7125, Novel_122, Novel_129, Novel_188, and Novel_201 were expressed at lower levels in RT-19 than in LT-19 samples (Fig. 3b), indicating that these five microRNAs were positively responsive to LT conditions. In contrast, miR156x, miR168a, Novel_68, Novel_88, Novel_90, Novel_177, Novel_195, Novel_198, and Novel_208 were more highly expressed in RT-19 than in LT-19 samples (Fig. 3b), indicating that these 9 microRNAs were negatively responsive to LT conditions. Therefore, LT can affect fruit senescence by mediating the expression of these 14 microRNAs that are responsive to LT conditions. In addition, qRT-PCR validation showed that the expression profiles of nine randomly selected microRNAs were positively correlated to those identified from the microRNAome data (*P* < 0.05; Figure S4), which indicates that the qRT-PCR results are consistent with the microRNAome data.

### Novel_188 represses *Pbr027651.1* expression and accelerates fruit senescence

**Fig. 5:**
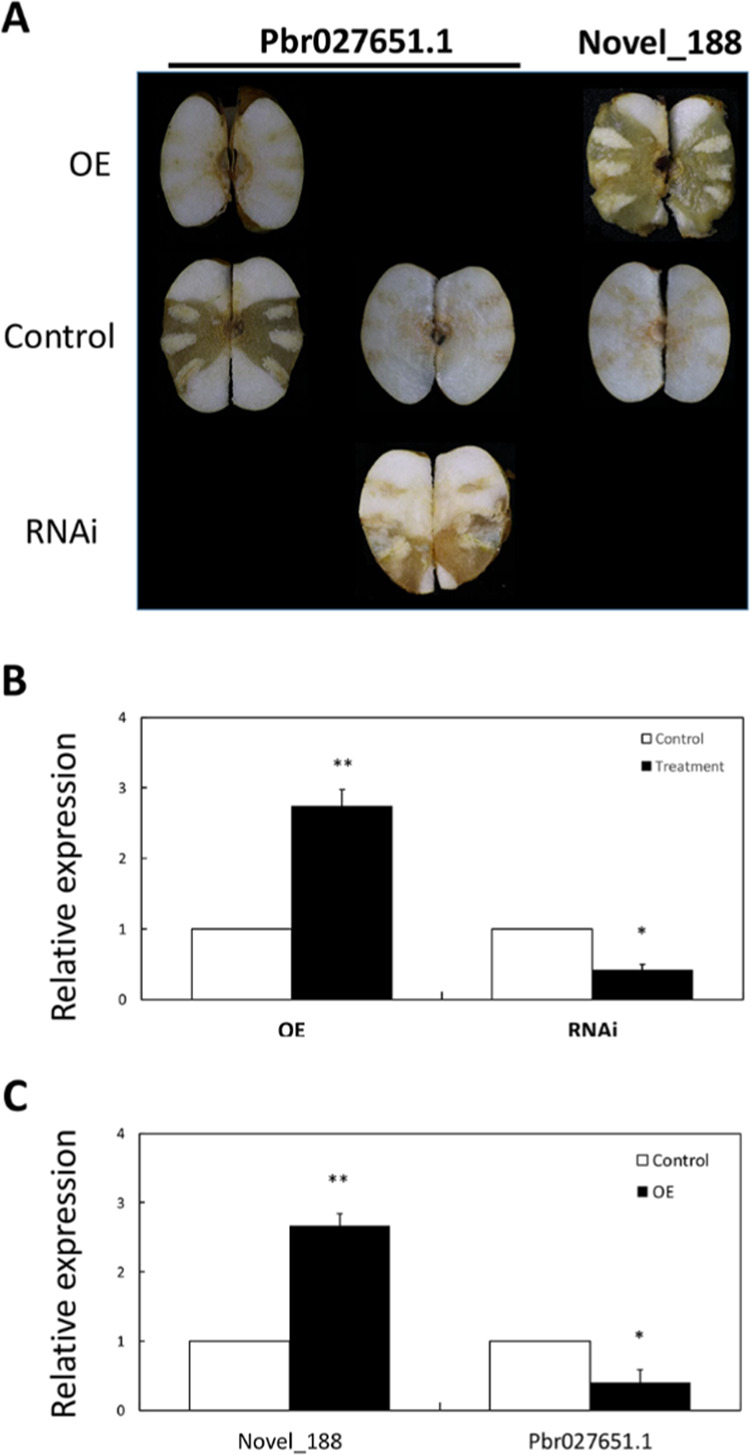
Transient transformation of Novel_188 and its target *Pbr027651.1*. **a** Senescence changes in transiently transformed pear fruits. **b** Expression levels of *Pbr027651.1* in fruits with overexpression and silencing of *Pbr027651.1*. The means and standard errors were compared using Student’s *t* test. Single and double asterisks indicate significant differences at *P* values of <0.05 and <0.01, respectively. **c** Expression levels of Novel_188 and *Pbr027651.1* in fruits overexpressing Novel_188. The means and standard errors were compared using Student’s *t* test. Single and double asterisks indicate significant differences at *P* values of <0.05 and <0.01, respectively

To confirm the roles of microRNA–mRNA interactions in fruit senescence, a pair of molecules, Novel_188 and *Pbr027651.1*, was selected for transient transformation of pear fruit. Fruit senescence was delayed (Fig. 5a) when *Pbr027651.1* was overexpressed in pear fruit (Fig. 5b). In contrast, fruit senescence was accelerated (Fig. 5a) when *Pbr027651.1* was silenced in pear fruit (Fig. 5b). This result shows that *Pbr027651.1* negatively mediates pear fruit senescence. Moreover, overexpressing Novel_188 accelerated fruit senescence (Fig. 5a), indicating that Novel_188 positively mediates pear fruit senescence. Furthermore, qRT-PCR analysis showed that Novel_188 was overexpressed but that *Pbr027651.1* was silenced in the transformed pear fruits (Fig. 5c). Therefore, Novel_188 can accelerate fruit senescence by repressing *Pbr027651.1* expression.

## Discussion

### Characterization of the mRNAs and compounds associated with fruit senescence under HT or LT conditions

**Fig. 6:**
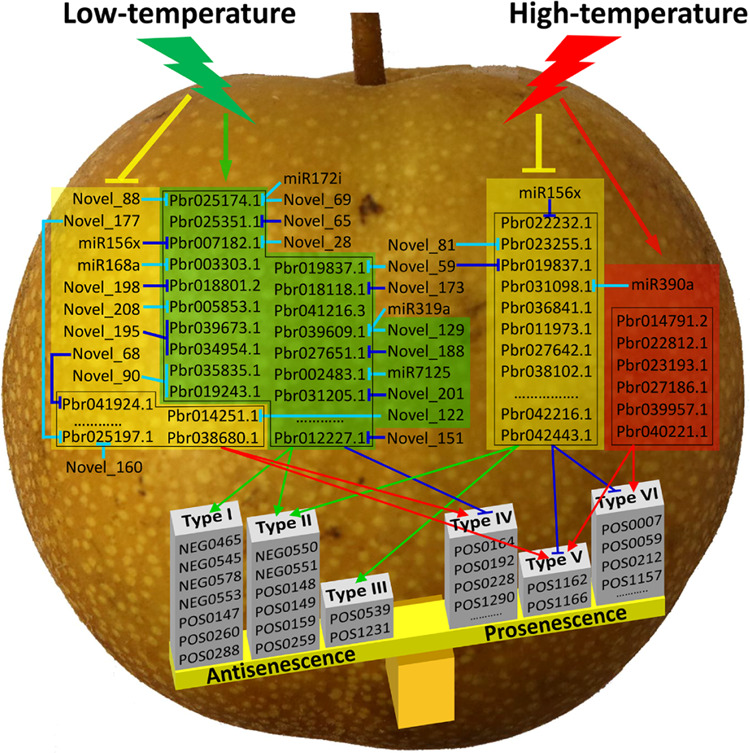
Diagram of molecular networks regulating fruit senescence under HT and LT conditions. The mRNAs and microRNAs with a yellow background are negatively responsive to HT or LT conditions. The mRNAs and microRNAs with red and yellow backgrounds are positively responsive to HT and LT conditions, respectively. The mRNAs and microRNAs without any background are not responsive to HT or LT conditions. Compared with RT, LT can affect the expression of 14 microRNAs and 530 mRNAs to promote the accumulation of antisenescent compounds of types I and II and to hinder the biosynthesis of prosenescent compounds of types IV and V, thus delaying fruit senescence. In contrast, HT can affect the expression of two microRNAs and 202 mRNAs to enhance the biosynthesis of prosenescent compounds of types V and VI and to hinder the accumulation of antisenescent compounds of types II and III, thus accelerating fruit senescence. Types I→VI are different types of metabolic compounds

Previously, fruit quality during postharvest storage has been widely studied using various treatments for delay of fruit senescence. These treatments can maintain the activities of fruit antioxidants (antisenescent metabolites) including carotenoids, ascorbic acid, glutathione, total phenolics, and total flavonoids^27–29^. Fruit senescence is determined by the balance of the antisenescent and prosenescent compounds that accumulate during postharvest storage. In this study, nontargeted metabolomics was conducted to survey the compounds associated with fruit senescence at three different temperatures. The results of this analysis, combined with the results of a correlation analysis between mRNAs and compounds, indicated that the compounds that are associated with fruit senescence under HT or LT conditions can be divided into six types (from I to VI; Table S10). Types I to III are antisenescent, and types IV to VI are prosenescent (Fig. 6). Notably, positive correlations were detected between two of the most common compounds in each type, and all six compounds of type II were positively correlated with six of the seven compounds of type I (Table S11). Moreover, the compounds of types II and V were associated with fruit senescence under both LT and HT conditions. The compounds of types I and IV were associated with fruit senescence under LT conditions, whereas the compounds of types III and VI were associated with fruit senescence under HT conditions.

Based on the above findings, the mRNAs associated with fruit senescence under HT or LT conditions were also identified. During fruit senescence under LT conditions, 444 LT-induced mRNAs were positively correlated with the compounds of types I and II and negatively correlated with the compounds of type IV, whereas 86 LT-repressed mRNAs were positively correlated with types IV and V. This result indicates that LT delays fruit senescence by enhancing the expression of mRNAs associated with the accumulation of antisenescent compounds and repressing the expression of mRNAs associated with the biosynthesis of prosenescent compounds. In contrast, during fruit senescence under HT conditions, six HT-induced mRNAs were positively correlated with the compounds of types V and VI, whereas 196 HT-repressed mRNAs were negatively correlated with the compounds of types V and VI and positively correlated with types II and III. This result suggests that HT accelerates fruit senescence by promoting the expression of mRNAs associated with the biosynthesis of prosenescent compounds and repressing the expression of mRNAs associated with the accumulation of antisenescent compounds. Notably, few mRNAs and compounds were associated with fruit senescence under both HT and LT conditions, indicating that the gene networks regulating fruit senescence under HT and LT conditions are different.

### Both HT and LT can affect fruit senescence via microRNA–mRNA interactions

In plants, microRNAs are derived from long single-stranded RNAs that can be folded to form stem-loop structures and then be cleaved by Dicer-like enzymes to form imperfectly double-stranded RNAs^30^. The mature microRNAs are loaded into silencing complexes including Argonaute proteins to degrade mRNAs by cleavage or to inhibit translation by combining target sites of coding regions and 3′ untranslated regions^31^. In this study, a dual-luciferase assay showed that microRNA–mRNA interactions occurred during fruit senescence under both HT and LT conditions (Fig. 4). Transient transformation showed that the LT-induced microRNA Novel_188 can mediate fruit senescence via microRNA–mRNA interactions to repress the expression of its target *Pbr027651.1* (Fig. 5). Therefore, the 27 isolated microRNAs are likely involved in fruit senescence under HT and/or LT conditions (Fig. 3).

Chromosomal location analysis showed that miR156x could not be mapped to the pear reference genome, whereas the other 26 microRNAs were located on 11 chromosomes and four scaffolds (Table S12). Among these 27 microRNAs, Novel_69 and Novel_88 belong to the miR172 family, and Novel_129, Novel_28, and Novel_59 belong to the miR159/miR319, miR156, and miR535 families, respectively; the other 17 novel microRNAs had relatively low identities with reported microRNAs (Table S12). In these families, miR172 regulates flowering time and is responsive to drought stress^32,33^. miR156 can be induced by heat stress^34^ and can increase salt and drought tolerance^35^. miR535 is induced by cold stress and negatively regulates cold tolerance^36^. miR168 is negatively responsive to salt and cold stresses^37^. Herein, the isolated microRNA miR168a was also repressed by LT conditions and may accelerate fruit senescence by interacting with its target along with the other six microRNAs (miR156x, Novel_195, Novel_198, Novel_208, Novel_88, and Novel_90; Fig. 6). Thus, LT can repress the expression of these seven microRNAs to enhance the expression of their targets. In contrast, LT can induce Novel_122 expression to reduce *Pbr014251.1* expression. Similarly, during fruit senescence under HT conditions, HT can induce miR390a expression to reduce *Pbr031098.1* expression (Fig. 6). These results suggest that either HT or LT affects fruit senescence by mediating the expression of microRNAs responsive to HT or LT conditions.

Moreover, it has been reported that miR159 is responsive to various stresses, including cold and heat^38^, and that it confers drought tolerance^39^. miR319, which is positively responsive to cold stress^40^ and enhances drought and salt tolerance^41^, regulates leaf senescence^42^. Novel_129, a member of the miR159 or miR319 family, was also induced by LT conditions. However, it is interesting that the Novel_129 target, *Pbr039609.1*, was also induced by LT conditions. Similar results were also detected for three other microRNAs (miR7125, Novel_188, and Novel_201) and their targets (*Pbr002483.1*, *Pbr027651.1*, and *Pbr031205.1*; Fig. 6). In contrast, LT repressed the expression of the two microRNAs Novel_68 and Novel_177 and their targets (*Pbr041924.1* and *Pbr025197.1*), and HT repressed the expression of both miR156x and its target *Pbr022232.1* (Fig. 6). This result may have occurred because microRNAs can have multiple targets (Table S13). For example, 14 mRNAs are targets of Novel_129, and five targets (*Pbr040785.1*, *Pbr006098.1*, *Pbr006097.1*, *Pbr041164.1*, and *Pbr040331.1*) had lower penalty scores for interaction than *Pbr039609.1*. In summary, both HT and LT can affect fruit senescence via microRNA–mRNA interactions.

### Molecular networks regulating fruit senescence under HT or LT conditions

Senescence is an unavoidable process in fruit life cycles and directly affects fruit quality, resistance to pathogens, and shelf life, contributing to the loss of economic value of postharvest fruits. LTs can effectively delay fruit senescence by retarding firmness loss and slowing metabolism during storage^8,9^. However, it is unclear how LTs affect pear fruit senescence. Herein, we found that LT can affect the expression of 530 mRNAs to promote the accumulation of antisenescent compounds of types I and II and to impede the biosynthesis of prosenescent compounds of types IV and V, contributing to the delay of fruit senescence (Fig. 6). In contrast, HT can affect the expression of 202 mRNAs to enhance the biosynthesis of prosenescent compounds of types V and VI and to inhibit the accumulation of antisenescent compounds of types II and III (Fig. 6), leading to the acceleration of fruit senescence. There are two potential pathways of mRNA expression affected by HT or LT conditions. First, either HT or LT can directly affect mRNA expression. Second, either HT or LT can affect mRNA expression by affecting microRNA–target interactions. During fruit senescence under HT conditions, HT directly affected miR156x and miR390a expression to disturb the expression levels of their targets (Pbr022232.1 and Pbr031098.1; Fig. 6). Similarly, LT directly affected the expression of 14 microRNAs to disturb the expression levels of their targets (Fig. 6). In these two networks, a relatively small number of mRNAs and compounds overlapped, and only miR156x was responsive to both HT and LT conditions. Thus, the molecular networks regulating fruit senescence under HT and LT conditions are different.

## Methods

### Plant materials

The pear cultivar Housui (cv. HS) was grown at Jiangpu Orchard, Nanjing Agricultural University (Nanjing, Jiangsu Province, China). Ripening fruits (RFs) of cv. HS were harvested on the 25th of August and divided into three groups. The first group contained 120 fruits that were stored at 40 ± 2 °C (HT). The second group contained 180 fruits that were stored at 25 ± 1 °C (RT). The third group contained 300 fruits that were stored at 4 ± 1 °C (LT). The fruit samples were collected at day 0 of storage and every 5 days from the start of storage to senescence. When over 30% of the stored fruits had decayed, the remaining fruits were regarded as senescent fruits. The fruits under LT treatment were initially sampled at 5-day intervals and then sampled every 20 days until senescence. Each sample contained flesh tissues from at least three fruits, and six samples were collected at each time point for each temperature treatment. The samples were immediately frozen in liquid nitrogen and stored at −80 °C until use.

### Nontargeted metabolomics

A total of 66 samples were selected for nontargeted metabolomics analysis by liquid chromatography–mass spectrometry; each sample was a biological replicate, and six replicates were selected. Acetonitrile and methanol were purchased from Merck (Darmstadt, Germany). The internal standard, DL-o-chlorophenylalanine, was purchased from GL Biochem (Shanghai) Ltd. (Shanghai, China; http://www.glbiochem.com/). The flesh samples were freeze dried and ground into a fine powder. One hundred milligrams of dry powder was extracted with 1.0 ml of 80% MeOH at 4 °C for 8 hours and then centrifuged at 10,000 × *g* for 10 min. The supernatant was filtered through a 0.22 mm filter and then run on an ACQUITY Ultra Performance Liquid Chromatography (UPLC) system (Waters, Massachusetts, USA) attached to an Agilent 6520 LC/MSD Ion Trap Mass Spectrometer (Agilent, Germany). Separation was performed on a 100 × 2.1 mm, 1.8 µm Waters ACQUITY UPLC HSS T3 (Waters) using the following gradient of acetonitrile versus 0.1% formic acid in water, run at 300 µl min^−1^ at 40 °C: 0 min, 5% acetonitrile; 2 min, 5% acetonitrile; 12 min, 95% acetonitrile; 15 min, 95% acetonitrile; 17 min, 5% acetonitrile; and 20 min, 5% acetonitrile. The levels of metabolic compounds were quantified via calculation of the peak areas and then standardization to standard curves. Statistical analysis of metabolite contents was performed with SPSS software version 19 (IBM, Chicago, Illinois) to distinguish the significantly differentially accumulated compounds between treatments (FDR < 0.05).

### Transcriptome sequencing and analyses

Total RNA was extracted from the 11 samples that were collected at 11 time points using an RNAprep Pure Plant Kit of Polysaccharides & Polyphenolics-rich (Tiangen, Beijing, China). The purity, concentration, and integrity were checked using a Nanophotometer spectrophotometer, a Qubit 2.0 Fluorometer, and an Agilent Bioanalyzer 2100 system (Agilent Technologies, Breda, Netherlands). The RNA samples that had high RINs (>7) were used for library construction and sequencing.

Before library construction, ribosomal RNAs were removed with an Epicentre Ribo-zero rRNA Removal Kit (Epicentre, USA). The remaining RNA was processed with an NEBNext Ultra Directional RNA Library Prep Kit for Illumina (NEB, USA). After cluster generation from the index-coded samples, the libraries were sequenced on an Illumina HiSeq 2000 platform (Illumina, San Diego, CA) as 150 bp paired-end reads. The raw data are available from the Sequence Read Archive (accession number: SRP233477).

After filtering the low-quality raw reads, the clean reads were mapped to the pear reference genome^43^ using Bowtie 2 v2.2.8 and HISAT2 v2.0.4.^44^ with an allowance of up to 3 bp mismatches. The mapped reads of each sample were assembled with StringTie v1.3.1^45^ through a reference-based approach.

### Differential expression and correlation analyses

The expression levels of microRNAs were calculated as transcripts per million-mapped reads, whereas the expression levels of mRNAs were calculated as fragments per kilobase of exon model per million-mapped fragments using Cufflinks v2.1.1^46^. Differential analyses of compounds, mRNAs, and microRNAs were performed based on a threshold of a log2(fold change) of 1. The correlation coefficients between compounds and mRNAs were calculated with SPSS software (IBM) and then corrected with a threshold FDR of 0.05.

### Small RNA sequencing and analysis

Small RNAs were size-selected from the total RNA of the 11 samples that were identical to the transcriptome analysis and were directly attached to specific adaptors for sequencing (Illumina). After synthesizing first-strand cDNA, PCR was performed with adaptor primers to enrich the target fragments, which were then sequenced using an Illumina HiSeq 2500/MiSeq (Illumina) as single-end 50 bp reads. The raw data for the small RNAs are available from the Sequence Read Archive (accession number: SRP233530).

The sequenced raw data were obtained by base calling and stored in FASTQ files, and the error rate was calculated by the Phred score (Qphred). After filtering the raw reads without adaptors, the reads with sequence-mutated adaptors, and the reads with lengths shorter than 18 bp or longer than 30 bp, the clean reads were mapped to the pear reference genome V1.0^43^ with Bowtie V2.0.6^47^ and TopHat v2.0.9^48^ with no mismatches allowed. Novel microRNAs were predicted using miRDeep-P with the default parameters as described in previous reports^49,50^. Moreover, these clean reads were BLASTed against miRBase (http://www.mirbase.org/) to annotate the microRNAs with high confidence^51^. The potential targets (mRNAs) of the detected microRNAs were evaluated by psRobot_tar in psRobot^52^.

### Dual-luciferase assay

The full-length sequences of pre-microRNAs were amplified from genomic DNA using high-fidelity KOD-Plus-Ver.2 DNA Polymerase (Toyobo, Osaka, Japan). The amplification products were inserted into pSAK277 vectors. The target sequences of microRNAs with sizes of 200–300 bp were amplified from first-strand cDNA. The amplification fragments were then inserted into a pGreenII Dual-Luciferase microRNA Target Expression Vector^18^. The subsequent process for introduction of the constructed vectors into *Agrobacterium tumefaciens*, suspension, infiltration, and culture of tobacco plants were identical to those in a previous study^18^. Luminescence assays were conducted using a Dual-Luciferase Reporter Assay System (Promega, Madison, WI). Firefly luciferase (Luc) and Renilla luciferase (Ren) activity levels were measured using a Cytation 3 Cell Imaging Multi-Mode Reader (BioTek, Santa Barbara, CA) for at least ten biological replicates for each assay. All primer sequences are listed in Table S14.

### Transient transformation of pear fruit

The full-length sequences of the Novel_188 precursors were amplified from genomic DNA, and the full-length sequence of *Pbr027651.1* was amplified from first-strand cDNA of pear fruits using KOD-Plus-Ver.2 high-fidelity DNA Polymerase (Toyobo). The amplification products were independently inserted into a pSAK277 vector under the control of the 35 S promoter. The process of transient transformation was identical to that in a previous report^53^. *Agrobacterium* containing Novel_188 precursors, *Pbr027651.1*, or an empty control vector were used to infiltrate 60 fruits. The full-length sequences and an ~400 bp fragment at the C-terminus of the target gene were amplified and inserted into the pTRV2 vector to construct a virus-induced gene silencing vector to induce gene silencing in pear fruit. The process of transient transformation was identical to that in a previous report^54^. Samples of flesh were collected from at least four infiltrated fruits, and six samples were collected for each construct at 10 days after injection . The samples were used for total RNA extraction and qRT-PCR analysis. All primer sequences are listed in Table S14.

### Quantitative real-time PCR testing

First-strand cDNA was synthesized using TransScript One-Step gDNA Removal and cDNA Synthesis Supermix (TransGen, Beijing, China). QRT-PCR was carried out in a LightCycler 480 II/96 Thermal Cycler (Roche Diagnostics, Rotkreuz, Switzerland). The reaction mixture and conditions were identical to those in a previous report^53^. The pear *TUBULIN* gene was used as an endogenous control^53^. The first-strand cDNA of microRNAs was synthesized using a miRcute miRNA First-Strand cDNA Synthesis Kit (TianGen, Beijing, China). qRT-PCR was performed with a miRcute Plus miRNA qRT-PCR Kit (TianGen) according to the manufacturer’s instructions, and the reaction mixture and conditions were identical to those in a previous report^18^. The pear 5 S gene was used as an endogenous control^18^. Moreover, a reaction mixture without cDNA template was used as a negative control. All primer sequences are listed in Table S14.

## Supplementary information

Supplementary figures

Supplementary tables

## Data Availability

The raw transcriptome and small RNA sequencing data have been deposited in the NCBI Sequence Read Archive database (https://www.ncbi.nlm.nih.gov/Traces/sra_ sub/sub.cgi) under the accession numbers SRP233477 and SRP233530.
