# Supplementary material for: Multiomics analyses unveil the involvement of microRNAs in pear fruit senescence under high- or low-temperature conditions
Source: Hortic Res. 2020 Dec 1;7:196. doi: 10.1038/s41438-020-00420-y (PMC7705739; doi:10.1038/s41438-020-00420-y)
Supplement: Supplementary file 1 — Supplementary figures [file 41438_2020_420_MOESM1_ESM.docx]

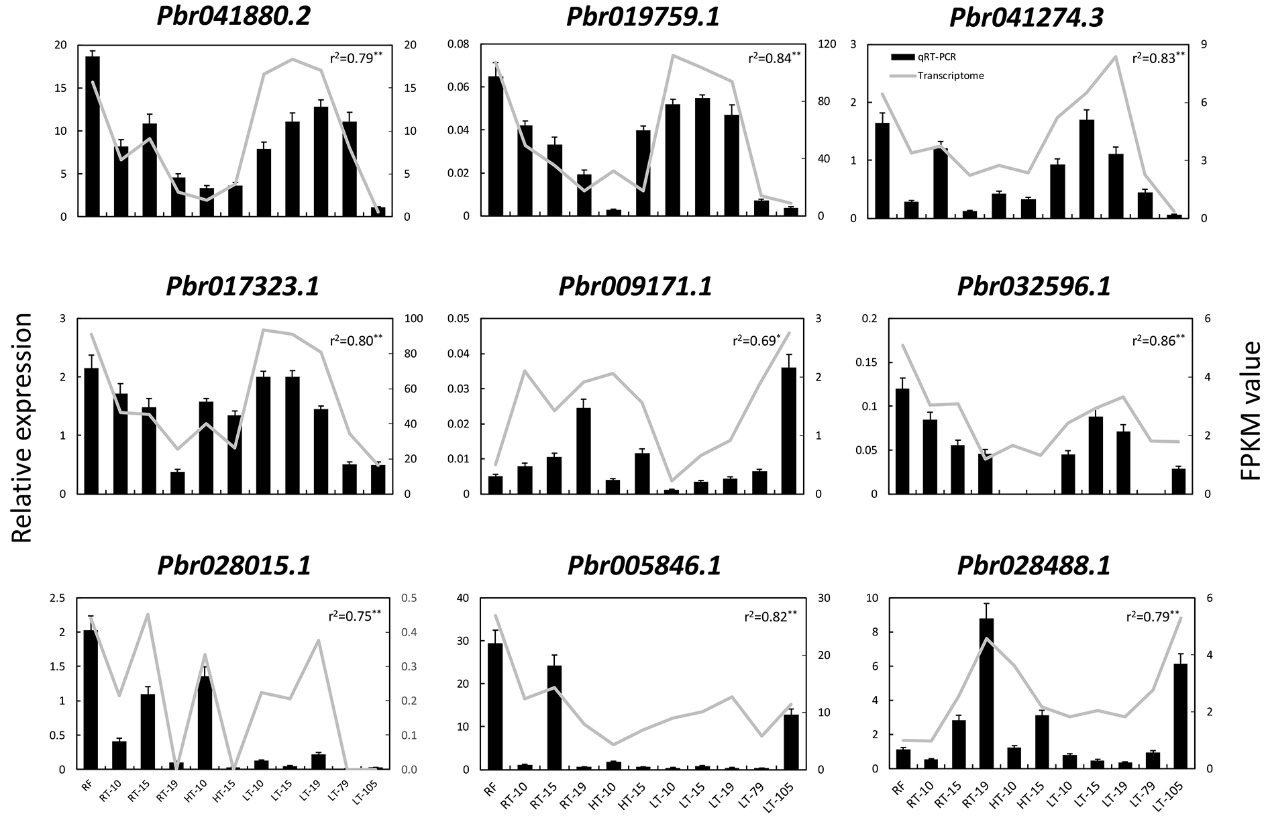


**Figure S1** qRT-PCR validation of the selected mRNAs in expression profiles. Pearson coefficient (r^2^) of qPCR and mRNAome data was calculated by SPSS software. Single and double stars indicate the significant correlation at *P*-value<0.05 and <0.01 levels, respectively.


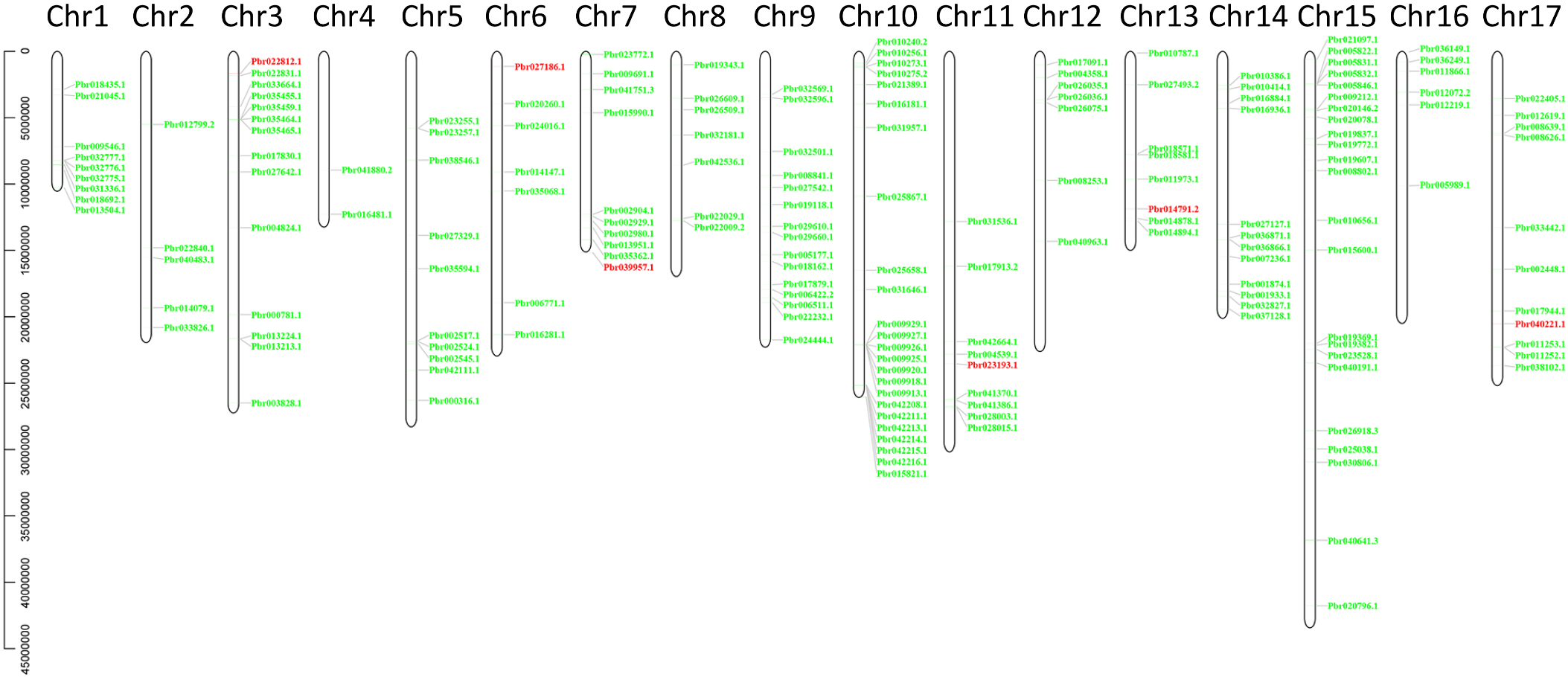
**Figure S2** Chromosome location of the mRNAs associated with fruit senescence under HT condition. The mRNAs that were positively responsive to fruit senescence under HT condition are signed by red color. The mRNAs that were negatively responsive to fruit senescence under HT condition are signed by green color.


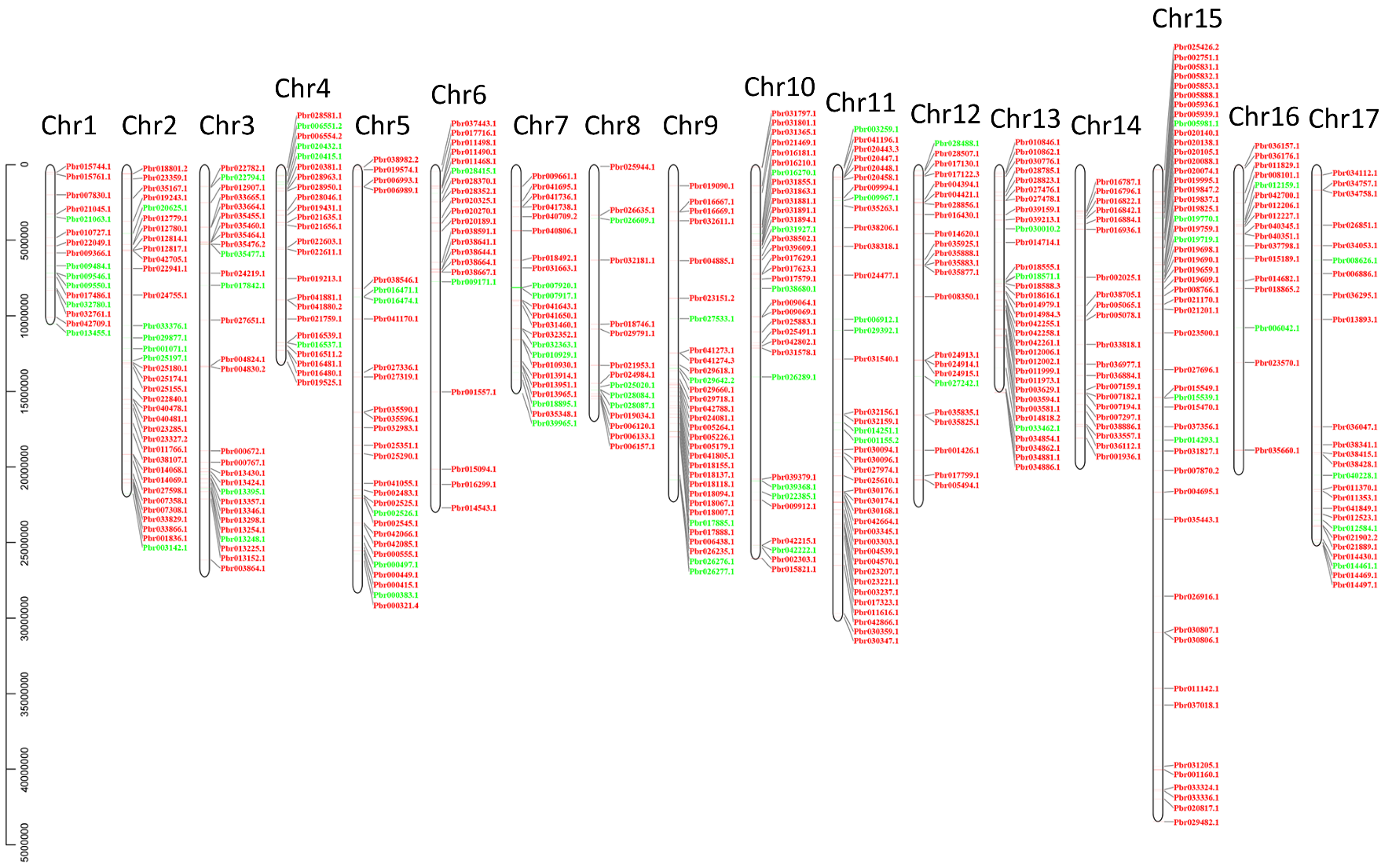


**Figure S3** Chromosome location of the mRNAs associated with fruit senescence under LT condition. The mRNAs that were positively responsive to fruit senescence under LT condition are signed by red color. The mRNAs that were negatively responsive to fruit senescence under LT condition are signed by green color.


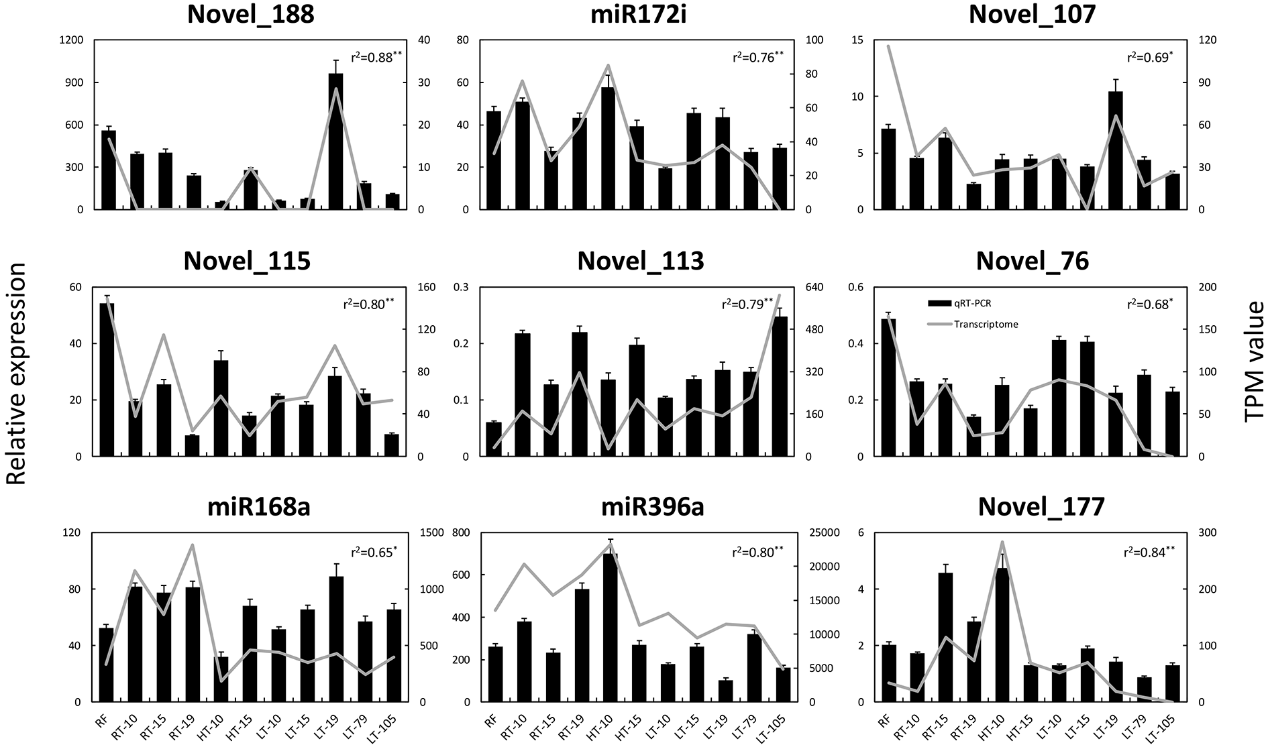


**Figure S4** qRT-PCR validation of the selected microRNAs in expression profiles. Pearson coefficient (r^2^) of qPCR and mRNAome data was calculated by SPSS software. Single and double stars indicate the significant correlation at *P*-value<0.05 and <0.01 levels, respectively.
